# Development and evaluation of deep learning models for estimating the organ at‐risk dose constraint from two‐dimensional cine magnetic resonance imaging scans during irradiation

**DOI:** 10.1002/acm2.70403

**Published:** 2025-11-27

**Authors:** Shohei Tanaka, Noriyuki Kadoya, Wingyi Lee, Hisamichi Takagi, Yoshiyuki Katsuta, Kazuhiro Arai, Yushan Xiao, Taichi Hoshino, Noriyoshi Takahashi, Keiichi Jingu

**Affiliations:** ^1^ Department of Radiation Oncology Tohoku University Graduate School of Medicine Sendai Japan; ^2^ Department of Radiological Technology School of Health Sciences Faculty of Medicine Tohoku University Sendai Japan

**Keywords:** 2D cine MRI, deep learning, dose prediction, MR‐Linac, radiotherapy

## Abstract

**Purpose:**

Two‐dimensional (2D) cine magnetic resonance imaging (MRI), available with a MR‐linear accelerator (MR‐Linac), allows real‐time visualization of anatomical information during irradiation. The present study aimed to develop and evaluate a deep learning model that can estimate the organ‐at‐risk (OAR) dose constraints (mainly bladder V37Gy) from 2D cine MRI.

**Methods:**

The present study enrolled 91 prostate cancer patients treated with MR‐Linac. From 381 treatment fractions, sagittal images at the start and end of the 2D cine MRI were extracted. Additionally, 3D MRI data acquired pre‐ and post‐irradiation were collected, from which bladder V37Gy was calculated. We designed the deep learning model to predict the end‐of‐irradiation bladder V37Gy value based on the bladder image on the end‐of‐irradiation 2D cine MRI. The model inputs included the start and end 2D cine MR images, a difference image between them, and the pre‐irradiation bladder V37Gy. The model output was the post‐irradiation bladder V37Gy. We utilized a five‐fold cross‐validation for model training and evaluated the performance using a test dataset. For reference, we also evaluated the predictions made using only the pre‐irradiation bladder V37Gy.

**Results:**

In the test dataset, the model‐predicted and true bladder V37Gy values showed a strong correlation (*r* = 0.89), with a mean absolute error (MAE) of 1.40 cm^3^. Using only the pre‐irradiation bladder V37Gy value yielded an *r* of 0.79 and an MAE of 2.02 cm^3^. Our model also achieved an area under the curve, sensitivity, and specificity values of 0.98, 0.91, and 0.95, respectively, in detecting dose constraint violations (bladder V37Gy of > 10 cm^3^).

**Conclusions:**

Our results demonstrated that deep learning can effectively predict the OAR dose constraints during irradiation. However, it is noteworthy that these results show only a limited improvement and are constrained by several limitations.

## 1 INTRODUCTION

In radiation therapy, adaptive radiation therapy (ART), which modifies the treatment plan according to the patient's anatomy on treatment day, has become the focus of active research recently.^1^, ^2^, ^3^ The advent of the Elekta (Stockholm, Sweden) Unity magnetic resonance (MR)‐linear accelerator (Linac), which integrates a 1.5T MR imaging (MRI) system with a 7 MV flattening filter‐free (FFF) linac, has enabled MR image acquisition during the treatment course. As a result, the application of MR‐guided ART is becoming increasingly widespread.^4^, ^5^, ^6^, ^7^, ^8^

In MR‐Linac‐based stereotactic body radiation therapy, the irradiation time is relatively long, lasting approximately 11 min.^9^ Such prolonged irradiation increases the likelihood of anatomical changes occurring during treatment. Particularly, in prostate cancer patients, anatomical changes can occur due to the presence of rectal gas, bladder filling, and muscle relaxation^6^, ^10^, ^11^ The MR‐Linac enables real‐time visualization using two‐dimensional (2D) cine MRI, a dynamic MRI technique that acquires 2D images of the target plane every 0.2 s. These images can be obtained in axial, sagittal, or coronal planes, allowing visualization of anatomical changes during irradiation. However, the accurate assessment of the dose distributions of organs‐at‐risk (OARs) remains challenging. Consequently, the anatomical changes during irradiation reportedly can result in higher doses to OARs than the planned doses.^12^, ^13^

To address these challenges, various approaches have been proposed to estimate the actual delivered dose using cine MRI. One such approach involves acquiring three‐dimensional (3D) cine MRI instead of 2D cine MRI for dose calculation. However, the current method for 3D cine MRI acquisition requires approximately 11‐second intervals, making it time‐consuming.^11^, ^14^ As an alternative, the use of deep learning to rapidly generate 3D images from 2D images has been proposed^15^ Nevertheless, this technique has not yet been adapted for 2D cine MRI. Furthermore, applying contouring and dose calculation to the generated or acquired 3D MRI during irradiation remains resource‐ and time‐intensive in clinical practice. Several studies have also reported the estimation of dose distributions using information from treatment log files and organ motions.^13^, ^16^, ^17^ However, these processes also remain resource‐intensive and time‐consuming, and online estimation of dose distributions during irradiation remains a considerable challenge.

As a new approach, we propose a deep learning‐based approach to estimate the dose constraints of OAR using 2D cine MRI acquired during irradiation. This approach aims to predict the OAR dose constraints that would result from delivering the entire treatment plan based on the current anatomical information captured by the 2D cine MRI. If successful, this method would allow the assessment of whether the OAR dose constraints are being met during irradiation, thereby supporting clinical decision‐making, including interrupting the beam, adjusting its position, or modifying the treatment plan. The present study first estimated the volume of the bladder receiving a dose of ≥37 Gy (bladder V37Gy) of < 10 cm^3^ as the primary OAR dose constraint,^18^ serving as an exploratory proof‐of‐concept. The estimation of this constraint is important because the bladder dose has been linked to urinary toxicity in hypofractionated prostate radiotherapy.^19^, ^20^ Some patients were found to exceed this constraint in the post‐irradiation dose assessment. Identifying such patients during irradiation would therefore have an important clinical value. Furthermore, the relationship between bladder volume and surface displacement is inherently nonlinear, with substantial variations among patients and between treatment fractions due to the differences in bladder filling. Additionally, the changes observed on 2D cine MRI occur gradually, making the accurate quantification of the displacement through visual inspection challenging. Therefore, we explored the potential of deep learning to capture such non‐linear variations in bladder volume and surface displacement across patients and treatment fractions, without relying on manual or mechanical displacement measurements.

Additionally, to comprehensively evaluate the estimation capabilities of the deep learning approach, we estimated not only the high‐dose regions in the bladder but also the medium‐dose regions and rectal doses, and assessed the predictive accuracy as a supplementary analysis.

The present study aimed to develop a deep learning regression model to estimate OAR dose constraints from the 2D cine MRI and to evaluate its predictive accuracy.

## 2 MATERIALS AND METHODS

### 2.1 Patient and treatment plan information

The present study included 91 prostate cancer patients (low‐risk: *n* = 27, moderate‐risk: *n* = 28, and high‐risk: *n* = 36) treated with the Unity MR‐Linac at our institution; all of whom had undergone 2D cine MRI. All patients had SpaceOAR (Boston Scientific Corporation, Marlborough, MA, USA) implantation. For low‐risk patients, the clinical target volume (CTV) was defined as the prostate alone, whereas for moderate‐ and high‐risk patients, the CTV included the prostate and seminal vesicles (up to 1.5 cm from the prostate base). The planning target volume (PTV) was created by adding a 3–5‐mm margin to the CTV. The bladder, rectum, femoral heads, and urethra were contoured as OARs. The prescribed dose was 36.25 Gy in five fractions for low‐ and moderate‐risk patients and 40 Gy in five fractions for high‐risk patients. Dose calculation was performed using a graphics processing unit (GPU)‐based Monte Carlo algorithm, with a grid size of 2.5–3 mm and an uncertainty of 1%. The treatment plans were generated using 7 MV FFF x‐rays and nine‐beam step‐and‐shoot intensity‐modulated radiotherapy. The dose constraints used for low‐ and moderate‐risk and for high‐risk patients at our hospital are summarized in Tables S1 and S2, respectively.

The study protocol, data management procedures, and scientific rationale were approved by the institutional ethics committee of our hospital. Accordingly, this study was conducted in accordance with the national guidelines and institutional regulations. Given that the present study was retrospective in nature involving no sample collection or intervention on human subjects, the requirement for patients’ informed consent was waived.

### 2.2 Study outline

**FIGURE 1:**
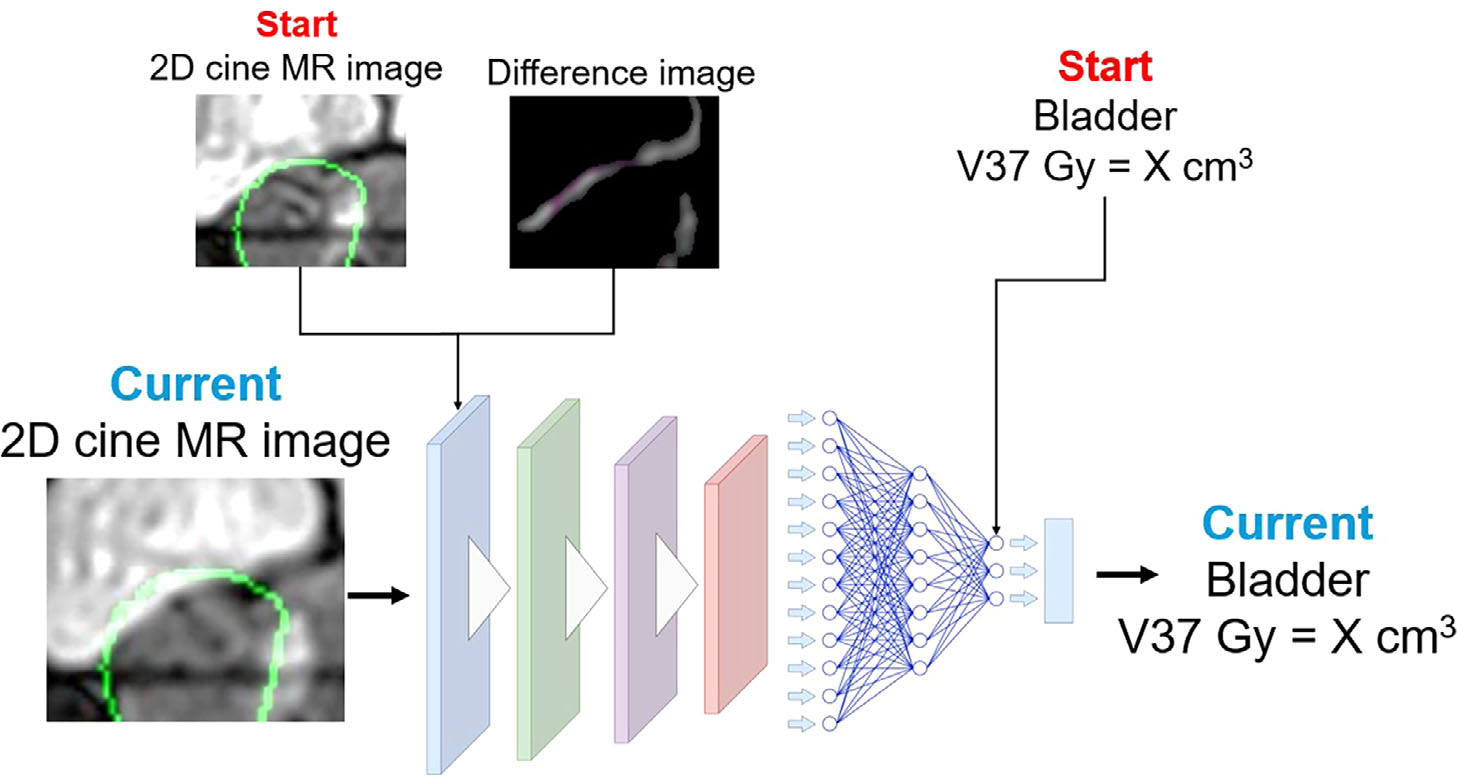
Conceptual diagram of the deep learning approach for predicting the volume of the bladder [in cm^3^] receiving a dose of ≥37 Gy (bladder V37Gy) based on the current two‐dimensional (2D) cine magnetic resonance imaging (MRI). As supplementary inputs, the model incorporated the 2D cine MRI at the start of irradiation (Start 2D cine MR image) and a difference image between the start and current images. Additionally, the numerical value of the bladder V37Gy at the start of irradiation (Start bladder V37Gy) was provided as a downstream input to the model.

A conceptual diagram of our study is shown in Figure 1. In our study, a single frame image extracted from the 2D cine MRI was defined as a “2D cine MRI image”. We estimated the current bladder V37Gy using deep learning based on the current 2D cine MR image of the bladder. In other words, the model predicts the bladder V37Gy assuming the entire treatment plan is delivered from start to finish, based on the current anatomical information of the bladder. Additionally, we hypothesized that incorporating information on the initial bladder V37Gy value at the start of irradiation and its subsequent anatomical changes would improve the prediction accuracy; thus, the 2D cine MRI at the start of irradiation (start 2D cine MR image) and a difference image were added upstream of the model, and the bladder V37Gy value at the start of irradiation (start bladder V37Gy) was added downstream of the model.

To correctly train the model, the current 2D cine MR image and the corresponding current bladder V37Gy should be correctly matched. Similarly, the start 2D cine MR image needed to match the start bladder V37Gy. In the present study, we used the end of the 2D cine MRI as the current 2D cine MR image, and the bladder V37Gy calculated using the 3D MR image acquired immediately after irradiation was used for the current bladder V37Gy. The start bladder V37Gy value was calculated using the 3D MR image acquired immediately before irradiation. Given that the bladder V37Gy during the mid‐irradiation phase was not available in the present study, the mid‐irradiation 2D cine MRI was not included in the model training. However, the trained model developed in the present study can also be applied to the mid‐irradiation 2D cine MRI frames to estimate the bladder V37Gy at that time. The following sections describe the dataset and model details.

### 2.3 Acquisition of 2D cine MRI and image preprocessing

The 2D cine MRI during irradiation were acquired using a balanced turbo field echo sequence. The axial and sagittal images were alternately acquired every 200 ms. The voxel size was 3.03 mm × 3.03 mm × 5.00 mm. For each fraction, 2D cine MRI were acquired in 91 patients. The 2D cine MRI were stored in nonreadable formats; therefore, the entire screen display was recorded using the AG‐Desktop Recorder (T. Ishii, Japan). Altogether, 405 recorded 2D cine MRI datasets were obtained, excluding fractions without recorded images. An example of the actual recorded 2D cine MRI is shown in Figure S1. The green line indicates the motion monitoring (MM) structure, which is used to monitor whether the target is within the irradiation region. This structure was defined to be the same as the PTV. Patients who had already exceeded the bladder dose constraint before irradiation were excluded, and 381 2D cine MRI were used for analysis. The sagittal and axial planes were acquired. However, the axial images did not capture the bladder and were excluded from the analysis; thus, only the sagittal images were used. Bladder filling predominantly causes superior and anterior displacements, rather than lateral movements.^21^ Additionally, prostate motion in the lateral direction is generally limited, whereas, in the anterior–posterior and cranial–caudal directions, more pronounced displacements are observed.^11^ Therefore, we hypothesized that a reasonable level of predictive accuracy could be achieved even when using sagittal‐plane images alone.

We captured the first and last frames at the start (start 2D cine MR image) and end (end 2D cine MR image) of the 2D cine MRI, respectively. A difference image was also created by subtracting the start 2D cine MR image from the end 2D cine MR image after both images were smoothed using a Gaussian filter. Additionally, supplementary frames taken 10 s after the start of irradiation (2D cine MR images after 10 s) were included. The 2D cine MR image after 10 s was paired with the start 2D cine MR image and used as an input for the deep learning model. This image pair was used to predict the bladder V37Gy value at the start of irradiation, in the absence of anatomical differences between the current and start 2D cine MR images. All 2D cine MR images after 10 s were visually confirmed to have no anatomical differences compared with the corresponding start 2D cine MR image. A difference image was also created by subtracting the start 2D cine MR image from the 2D cine MR image after 10 s using the same process.

To prepare these images as inputs for the deep learning model, the following preprocessing steps were applied. First, image cropping was performed only on regions relevant to the bladder dose. The detailed image cropping methods are shown in Table S3. Second, all images were normalized by dividing them by the maximum value of the training dataset described in detail later. Third, noise was reduced by removing pixels with signal intensities of < 0.2 or regions smaller than 50 pixels from the difference images. Figure S2 shows the preprocessed 2D cine MR image used as input to the deep learning model.

### 2.4 Extraction of bladder V37Gy

Almost all patients underwent MR‐guided radiation therapy using the adapt‐to‐shape workflow, wherein the treatment plan is optimized based on the patients’ anatomical information on the treatment day to ensure dose distribution consistent with the patients’ daily anatomy. In the treatment workflow, all patients underwent 3D T2‐weighted MRI scans at the following three time points: before treatment (pre‐MRI), immediately before irradiation (position verification [PV]‐MRI), and immediately after irradiation (post‐MRI). Pre‐MRI refers to the first MRI performed on treatment day, which is utilized to create a treatment plan. PV‐MRI is the MRI conducted immediately before irradiation to confirm whether the target is correctly positioned within the PTV. Post‐MRI is conducted immediately after irradiation to assess whether any movement of the target or OARs occurred during irradiation.

The pre‐MRI OARs structures were transferred to the PV‐ and post‐MRIs using deformable image registration and then slightly adjusted by medical physicists. For MRI‐based dose calculation, the relative electron density was assigned using the following methods: a patient‐specific method, in which the mean relative electron density for each structure was calculated from the corresponding computed tomography and assigned to the MRI; and a population‐based Hounsfield unit assignment method^22^ in which representative electron densities were assigned to each structure based on the data from a patient cohort. The treatment plan was transferred to the PV‐ and post‐MRIs, and the dose was recalculated with fixed segments to obtain the bladder V37Gy values. The V37Gy calculated from the PV‐MRI was used as the input for the deep learning model, representing the bladder dose at the start of irradiation. The V37Gy calculated from the post‐MRI was used as the model output (ground truth), representing the bladder dose at the end of irradiation. Hereafter, the post‐MRI bladder V37Gy is defined as the true bladder V37Gy. Altogether, 12.9% of all post‐MRI treatment fractions did not meet the dose constraint of bladder V37Gy of < 10 cm^3^.

### 2.5 Model architecture and training setting

**FIGURE 2:**
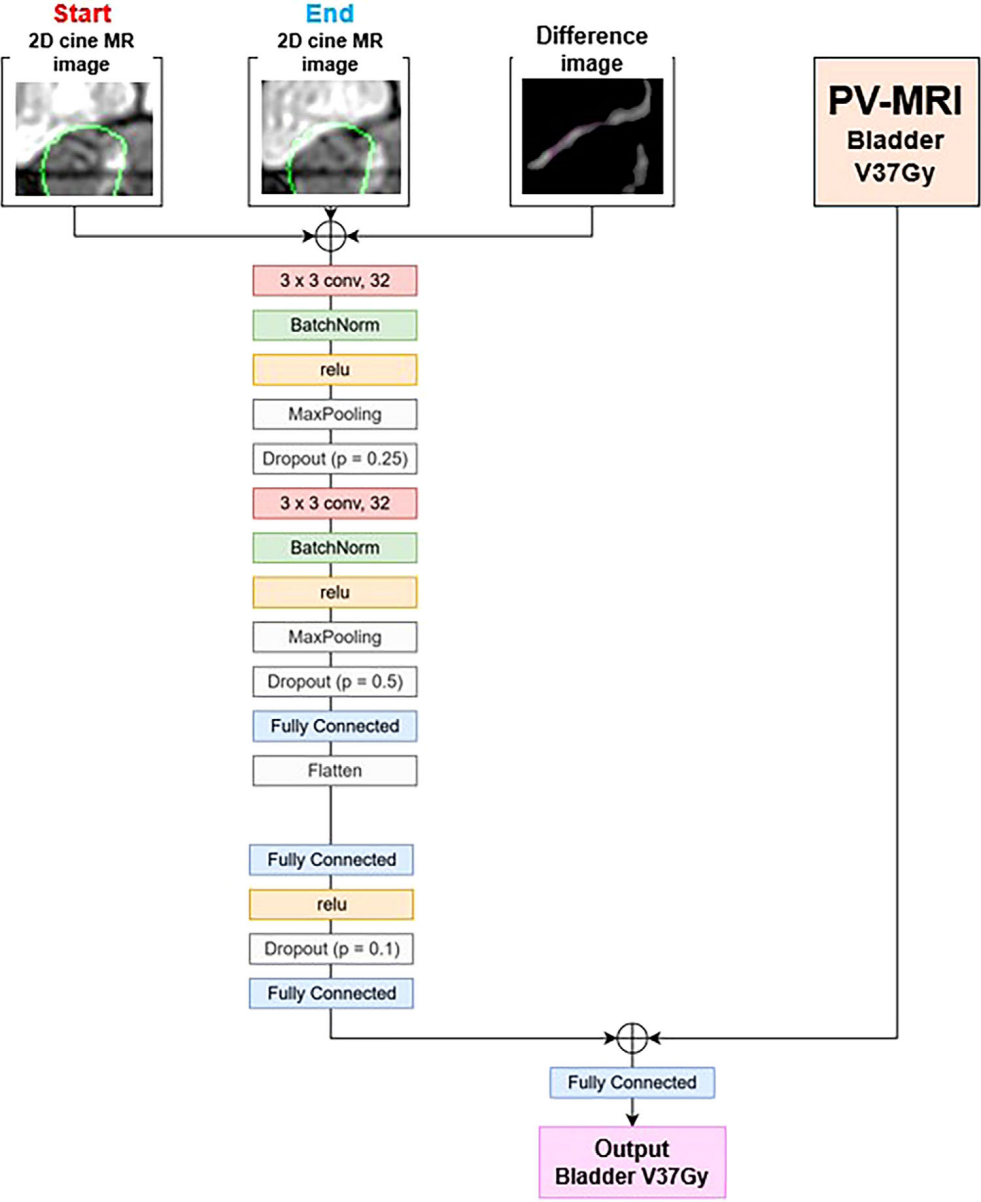
Architecture of the main deep learning model for predicting the volume of the bladder [cm^3^] receiving ≥37 Gy (bladder V37Gy) from two‐dimensional (2D) cine magnetic resonance imaging (MRI). The model takes 2D cine MR image and bladder V37Gy at the beginning of irradiation as inputs and outputs of the predicted bladder V37Gy at the end of irradiation. This model was used as the main model (Model 1) in this study.

The architecture of the main model (Model 1) is shown in Figure 2. The inputs to the model included the start 2D cine MR image, end 2D cine MR image, difference image, and PV‐MRI bladder V37Gy. In this configuration, the model was trained to predict the true bladder V37Gy. In another configuration, the start 2D cine MR image, 2D cine MR image after 10 s, difference image, and PV‐MRI bladder V37Gy were simultaneously used as inputs. In this case, the model was trained to predict the PV‐MRI bladder V37Gy.

The upstream section included two convolutional layers, two activation functions, two max pooling layers, two dropout layers with probability of 0.25 and 0.5, and one fully connected layer. The downstream included two fully connected layers, one activation function, and one dropout layer with probability of 0.1. The PV‐MRI bladder V37Gy was concatenated with the downstream layers’ output. A regression layer was used as the final output layer. Further details on the model dimensions are shown in Table S4. The model architecture was designed using the rationale described below. In the upstream section, the start and end 2D cine MR images, along with the difference image, were used to extract the bladder shape and its morphological changes. In the downstream section, the PV‐MRI bladder V37Gy was incorporated to provide information on the bladder V37Gy at the beginning of the irradiation.

The model was implemented and trained using MATLAB R2024b (MathWorks, Natick, MA, USA). The training was conducted with a maximum of 200 epochs, a mini‐batch size of 64, an adaptive moment estimation optimizer, an initial learning rate of 0.001, and an L2 regularization factor of 0.01. Validation was performed every 64 iterations, and early stopping was applied if no improvement was observed in the validation performance with over 100 consecutive validations. The loss function used was the half mean squared error. Model training was performed using a GPU (RTX A6000, NVIDIA).

### 2.6 Training and test process of the deep learning model

**FIGURE 3:**
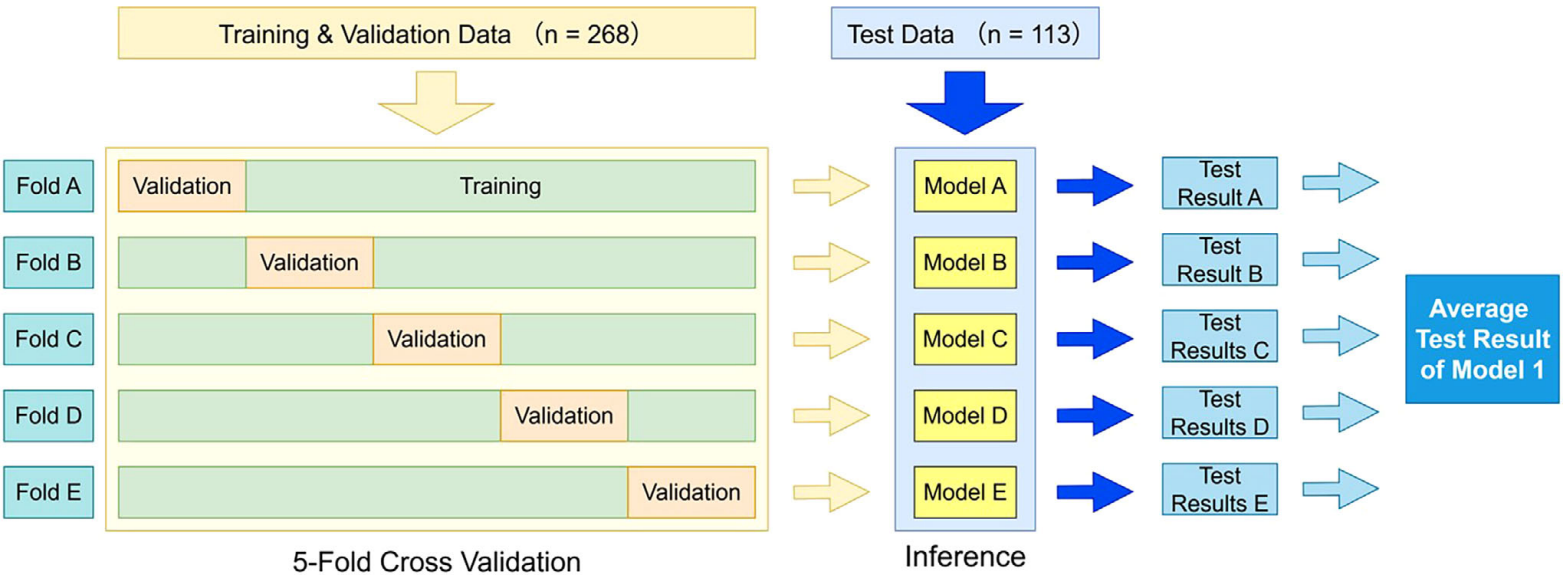
The training, inference, and evaluation methods of the deep learning model using the training, validation, and test datasets. Training of the deep learning model were performed using five‐fold cross‐validation. Five trained models were created, and the test dataset was evaluated using each model. The final prediction performance was obtained by averaging the results obtained from the five models. This entire process was performed separately for each model architecture.

Figure 3 shows the model training and evaluation methods using the training, validation, and test datasets. Our study referred to the training and evaluation methods used in previous deep learning studies.^23^, ^24^, ^25^ Of the total 381 data, 70% were randomly assigned to the training and validation datasets (*n* = 268, from 54 patients), whereas the remaining 30% (*n* = 113, from 37 patients) were assigned to the test dataset. The split maintained an approximately constant ratio of bladder dose constraint violations and compliances. The training and validation datasets were used for five‐fold cross‐validation. The data from the patients included in the test dataset group were not used in the training or validation datasets. Data augmentation was applied to the training dataset. The details of the image and numerical data augmentation methods are provided in Table S5. To train the model to effectively identify the patients exceeding the bladder V37Gy constraint, data from the patients who exceeded the constraint were augmented, resulting in an approximately equal number of over‐ and under‐constraint patients. Subsequently, the entire training set was further augmented ten‐fold to increase the data volume. The test dataset was input into five independently trained models obtained through five‐fold cross‐validation. Final performance of Model 1 was calculated by averaging the five test results, which then produced a single test result.

### 2.7 Comparison models

**FIGURE 4:**
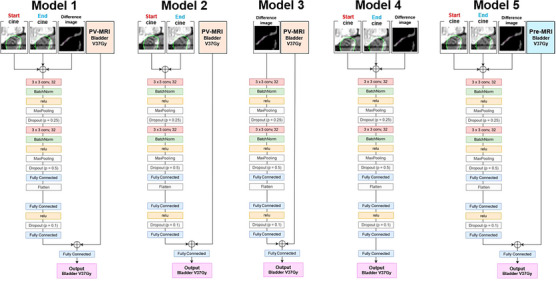
Architectures of the main model (Model 1) and the other four comparative models for predicting the volume of the bladder [cm^3^] receiving ≥37 Gy (bladder V37Gy) from two‐dimensional (2D) cine magnetic resonance imaging (MRI). Model 1 corresponds to the same architecture shown in Figure 2. Model 2 used only the 2D cine MR images acquired at the start and end of irradiation as inputs. Model 3 used only the difference image between the start and end 2D cine MR images. Model 4 excluded the position verification (PV) MRI–based bladder V37Gy. Model 5 replaced the PV‐MRI bladder V37Gy with the pre‐MRI bladder V37Gy.

To evaluate the usefulness of architecture of Model 1, four other models were prepared for comparison. The architectures of these models are shown in Figure 4. The comparative models, which were derived from Model 1 by modifying the input data, were as follows: Model 2 used only the start and end 2D cine MR images; Model 3 used only the difference image between the start and end 2D cine MR images; Model 4 excluded the PV‐MRI bladder V37Gy; and Model 5 replaced the PV‐MRI bladder V37Gy with the pre‐MRI bladder V37Gy (Figure 4). Additionally, for reference, we evaluated a case with the PV‐MRI bladder V37Gy value directly used as a predictor without using any deep learning. All the models were trained and evaluated under the same conditions and methods as Model 1.

### 2.8 Prediction using complex model architecture

Simple model architectures were used in the present study. However, to investigate whether model complexity and the number of parameters could improve the predictive accuracy, the following two additional models were employed: NasNetLarge^26^ (parameters: 88.9 million) and Residual Neural Network^27^ with 101 layers (ResNet101) (parameters: 44.6 million). The input structure was modified to accept three images instead of one, and the final fully connected layer was concatenated with the PV‐MRI bladder V37Gy input. The output layer was also changed from a classification to a regression layer. These models were trained and evaluated under the same conditions and methods as Model 1.

### 2.9 Prediction using 2D cine MRI during irradiation

Although the present study primarily predicted the post‐MRI bladder V37Gy value, additional analyses were performed using 2D cine MR images acquired during irradiation for three test patients whose true bladder V37Gy value exceeded 10 cm^3^ and exhibited small prediction errors (absolute error = 0.11–0.29 cm^3^). Specifically, the 2D cine MR images acquired during irradiation were input into Model 1, and the trends of predicted values of Model 1 during irradiation were evaluated. This evaluation aimed to determine whether the model's bladder V37Gy predictions were transient and fluctuating or consistently reflected the temporal anatomical changes of the bladder throughout the irradiation process.

### 2.10 Prediction of additional OAR dose constraints

Although the main focus of the present study was the prediction of bladder V37Gy, we additionally predicted the bladder V18.1 Gy (cm^3^) value to evaluate the model's performance for medium‐dose constraints. For bladder V18.1 Gy predictions, the same input images used for bladder V37Gy were employed. Using the architecture utilized in Model 1, the inputs consisted of the start and end 2D cine MR images, the difference image, and PV‐MRI bladder V18.1 Gy. The model was trained to perform regression for true bladder V18.1 Gy predictions. The same patients in the training and testing datasets used for predicting the bladder V37Gy values were employed to evaluate this model.

To evaluate the predictive accuracy for another OAR, the rectum, we also predicted the dose constraints of rectum V29Gy (cm^3^) and rectum V18.1 Gy (cm^3^). The architecture of the model based on Model 1, which was used to predict the rectal dose constraints, is shown in Figure S3. Axial and sagittal images were used as the input images, with the cropping method described in Table S3. The other image preprocessing procedures were identical to those used for the bladder. The model inputs consisted of the start and end 2D cine MR images, the difference image, and either the PV‐MRI rectum V18.1 Gy or PV‐MRI rectum V29Gy. The model performed regression prediction of the true rectum V18.1 Gy or rectum V29Gy. The same test dataset used for predicting the bladder V37Gy value was employed to evaluate this model. The patients in the training and testing datasets were the same as those used for bladder V37Gy; however, treatment fractions with rectal gas observed on 2D cine MRI and those with a V29Gy volume of zero were excluded from the dataset.

### 2.11 Evaluation

To evaluate the agreement between the predicted and true values, we calculated the Spearman's correlation coefficient, root mean squared error (RMSE), and mean absolute error (MAE) for all dose constraints. For bladder V37Gy, to assess the model's ability to detect cases exceeding the dose constraint, we evaluated the area under the curve (AUC), sensitivity, and specificity, which were calculated by defining bladder V37Gy of > 10 cm^3^ as positive. Additionally, to identify which patient feature was associated with larger errors on the prediction of bladder V37Gy for Model 1, we performed a subgroup analysis of the predictive accuracy. In the test dataset, patients were divided into two groups based on the following factors: bladder volume on PV‐MRI, bladder volume on post‐MRI, volume of bladder filling from PV‐ to post‐MRI, prostate risk group, movement of the prostate in the superior, inferior, anterior, or posterior directions from PV‐ to post‐MRI, and rectal gas. For each factor, the absolute differences in prediction errors between the two groups were calculated and statistically compared.

Statistical significance was assessed using the *t*‐test, with a *p*‐value of < 0.05 considered statistically significant.

## 3 RESULTS

The Pre‐, PV‐, and post‐MRI bladder volumes were 117.9 ± 59.0, 150.6 ± 73.1, and 205.5 ± 101.2 cm^3^, respectively. The absolute differences in bladder V37Gy between pre‐ and PV‐MRI and between PV‐ and post‐MRI were 1.81 ± 1.69 and 1.86 ± 2.30 cm^3^, respectively, for the training dataset and were 1.68 ± 1.70 and 2.02 ± 2.35 cm^3^, respectively for the test dataset.

Table 1 presents the Spearman's correlation coefficient, RMSE, and MAE for Model 1 and bladder V37Gy using solely PV‐MRI data. In the test dataset, the comparison of the performance of Model 1 and PV‐MRI bladder V37Gy showed Spearman's correlation coefficients of 0.89 and 0.79, MAE of 1.40 and 2.02 cm^3^, RMSE of 1.82 and 2.65 cm^3^, and AUC of 0.98 and 0.93, respectively. All metrics indicated that Model 1 achieved a significantly higher predictive accuracy (*p* < 0.001), although the degree of improvement was limited.

**TABLE 1:** Spearman's correlation coefficient, RMSE, and MAE between the predicted and true bladder V37Gy in the training and test datasets for Model 1 and PV‐MRI bladder V37Gy.

		Model 1	PV‐MRI bladder V37Gy	*p*‐value
Training	Spearman's correlation coefficient	0.99 ± 0.00	0.80 ± 0.02	< 0.001
	MAE (cm^3^)	0.32 ± 0.03	2.76 ± 0.05	< 0.001
	RMSE (cm^3^)	0.42 ± 0.03	3.58 ± 0.12	< 0.001
	AUC	1.00 ± 0.00	0.90 ± 0.02	< 0.001
Test	Spearman's correlation coefficient	0.89 ± 0.01	0.79 ± 0.00	< 0.001
	MAE (cm^3^)	1.40 ± 0.05	2.02 ± 0.00	< 0.001
	RMSE (cm^3^)	1.82 ± 0.06	2.65 ± 0.00	< 0.001
	AUC	0.98 ± 0.00	0.93 ± 0.00	< 0.001

Abbreviations: PV: position verification, V37Gy: volume of the bladder [in cm3] receiving a dose of ≥37 Gy, MAE: mean absolute error, RMSE: root mean squared error, AUC: area under the curve.

Table 2 shows the Spearman's correlation coefficient, RMSE, and MAE between the predicted and true bladder V37Gy values for each model. In the training datasets, the Spearman's correlation coefficients for Models 1, 2, 3, 4, and 5 were all 0.99. However, in the test dataset, the Spearman correlation coefficients for Models 1 through 5 were 0.89, 0.89, 0.84, 0.75, and 0.83, respectively, with Model 1 demonstrating the high correlation with the true bladder V37Gy. Model 1 also achieved the low MAE and RMSE among the models in the test dataset. However, no significant difference in the Spearman's correlation coefficient, MAE, or RMSE was noted between Models 1 and 2. Table 2 also shows the AUC, sensitivity, and specificity values of the training and test datasets. In the training datasets, the AUCs for Models 1 through 5 were all 1.00, showing no differences among the models. In the test dataset, the AUCs were 0.98, 0.97, 0.96, 0.94, and 0.92, respectively, with Model 1 showing the highest performance. The sensitivity values varied more notably among all models, with Model 1 achieving the highest value (0.91). However, no significant differences in the AUC, sensitivity, and specificity values were observed between Models 1 and 2.

**TABLE 2:** Spearman's correlation coefficient, MAE, RMSE, AUC, sensitivity, and specificity between the predicted and true bladder V37Gy in the training and test datasets for each model.

		Model 1	Model 2	Model 3	Model 4	Model 5	*p*‐value (Model 1 vs. Model 2)	p value (Model 1 vs. Model 3)	p value (Model 1 vs. Model 4)	p value (Model 1 vs. Model 5)
Training	Spearman's correlation coefficient	0.99 ± 0.00	0.99 ± 0.00	0.99 ± 0.00	0.99 ± 0.00	0.99 ± 0.09	0.943	0.529	0.009	0.014
	MAE (cm^3^)	0.32 ± 0.03	0.35 ± 0.07	0.32 ± 0.03	0.51 ± 0.12	0.46 ± 0.04	0.303	0.217	0.058	0.003
	RMSE (cm^3^)	0.42 ± 0.03	0.47 ± 0.09	0.45 ± 0.03	0.63 ± 0.13	0.60 ± 0.05	0.258	0.002	0.046	0.004
	AUC	1.00 ± 0.00	1.00 ± 0.00	1.00 ± 0.00	1.00 ± 0.00	1.00 ± 0.00	0.138	0.086	0.246	0.106
	Sensitivity	0.95 ± 0.03	0.90 ± 0.03	0.95 ± 0.03	0.86 ± 0.06	0.87 ± 0.03	0.186	0.972	0.093	0.057
	Specificity	0.99 ± 0.00	0.99 ± 0.00	0.99 ± 0.00	1.00 ± 0.00	1.00 ± 0.00	0.657	0.023	0.166	0.054
Test	Spearman's correlation coefficient	0.89 ± 0.01	0.89 ± 0.00	0.84 ± 0.01	0.75 ± 0.03	0.83 ± 0.02	0.176	0.001	< 0.001	0.002
	MAE (cm^3^)	1.40 ± 0.05	1.37 ± 0.04	1.63 ± 0.07	2.16 ± 0.12	1.82 ± 0.08	0.121	0.002	< 0.001	0.001
	RMSE (cm^3^)	1.82 ± 0.06	1.82 ± 0.03	2.12 ± 0.07	2.74 ± 0.16	2.38 ± 0.11	0.834	< 0.001	< 0.001	< 0.001
	AUC	0.98 ± 0.00	0.97 ± 0.01	0.96 ± 0.02	0.94 ± 0.02	0.92 ± 0.02	0.165	0.096	0.011	0.006
	Sensitivity	0.91 ± 0.09	0.83 ± 0.03	0.77 ± 0.12	0.52 ± 0.25	0.55 ± 0.10	0.109	0.022	0.011	< 0.001
	Specificity	0.95 ± 0.01	0.96 ± 0.02	0.94 ± 0.01	0.96 ± 0.02	0.94 ± 0.02	0.208	0.426	0.512	0.109

Abbreviations: MAE: mean absolute error, RMSE: root mean squared error, AUC: area under the curve, V37Gy: volume of the bladder [in cm3] receiving a dose of ≥37 Gy.

**FIGURE 5:**
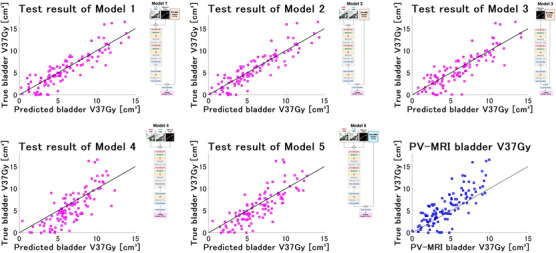
Correlation between the predicted and true bladder V37Gy (volume of the bladder receiving ≥ 37 Gy) values when using the main deep learning model (Model 1), other four comparative models (Model 2–5), and position verification (PV)‐MRI bladder V37Gy in the test dataset. The predicted bladder V37Gy represents the average results from the five‐fold cross‐validation. The architecture of the deep learning model used for prediction is shown to the right of the correlation plot. The lines matching the predicted and true bladder V37Gy values are shown as black straight lines.

Figure 5 shows the correlation between the predicted and true bladder V37Gy values for each model in the test dataset. The coefficients of determination (*R* ^2^), indicating linearity, were 0.79, 0.79, 0.73, 0.60, and 0.68 for Models 1 to 5, respectively. Models 1 and 2 showed better linearity as compared to Models 3, 4, and 5 with smaller deviations between the predicted and true values. Model 1 showed small prediction errors, particularly in the region where the bladder V37Gy value was < 10 cm^3^. The R^2^ value of the regions with a bladder V37Gy value of < 10 cm^3^ for Model 1 was 0.68, whereas the that for regions with a bladder V37Gy value of > 10 cm^3^ was 0.13. The PV‐MRI bladder V37Gy values are shown as a reference, with an R^2^ value of 0.65. This indicates that the linearity of Models 1 and 2 was better than that of the PV‐MRI bladder V37Gy.

**FIGURE 6:**
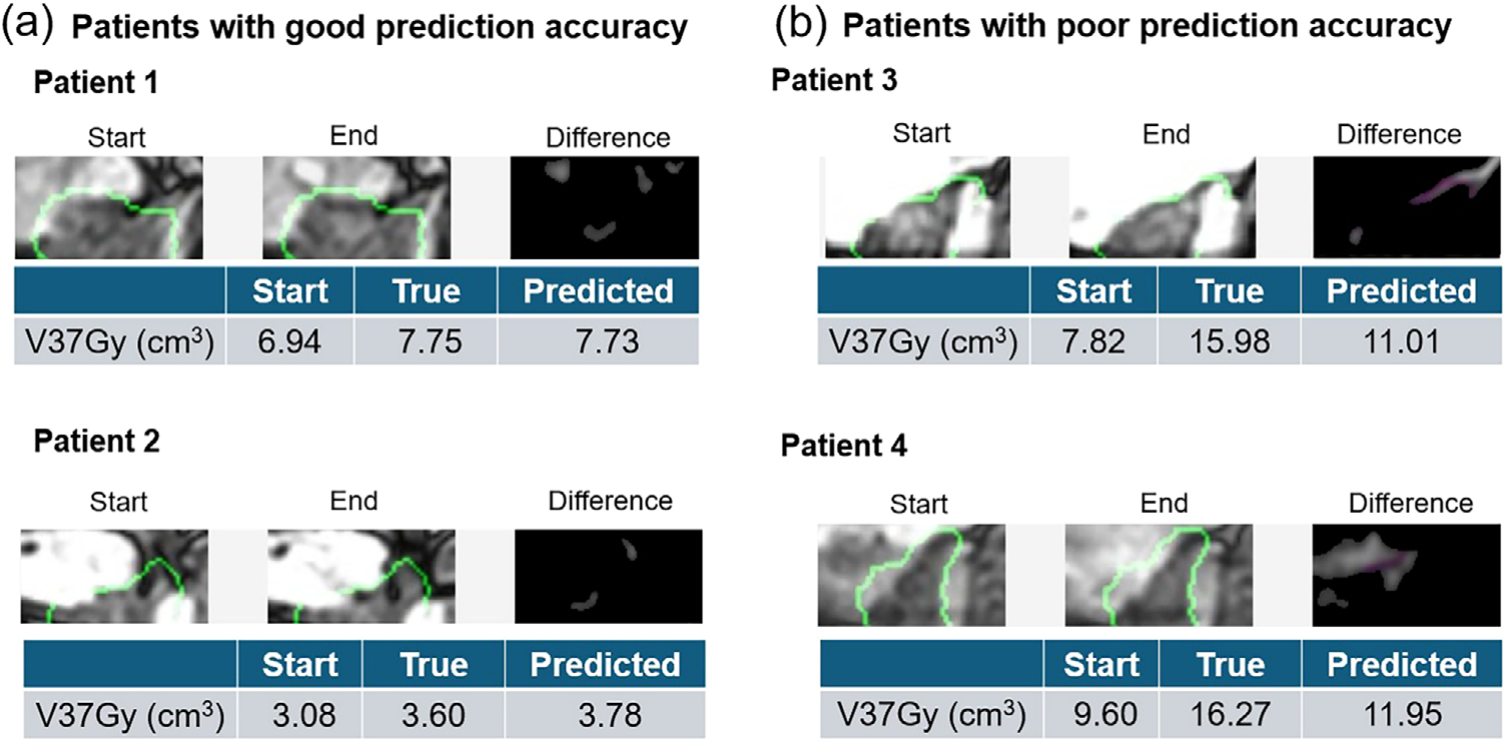
Examples of (a) good vs (b) poor predictions, showing two‐dimensional (2D) cine magnetic resonance (MR) image, difference image, and corresponding true/predicted bladder V37Gy values (volume of the bladder receiving ≥ 37 Gy). The sagittal images of a prostate cancer patient are shown. For each patient, the start and end 2D cine MR images and difference image between these images are presented. Additionally, the bladder V37Gy at the start of the irradiation, true bladder V37Gy, and predicted bladder V37Gy by Model 1 (main model) are also shown.

Figure 6 shows typical patients with good and poor prediction accuracies in Model 1. In the two patients with good prediction accuracy, the difference images appeared almost entirely black, indicating minimal differences between the start and end of the 2D cine MR images. In these cases, the true bladder V37Gy values were 7.75 and 3.60 cm^3^, whereas the predicted values were 7.73 and 3.78 cm^3^, respectively, showing a good agreement. Contrarily, the two patients with poor predictions involved bladders that were substantially filled, where even small anatomical changes in 2D cine MR image led to large variations in bladder V37Gy. Additionally, for Patient 4, the difference in bladder signal intensity between the start and end 2D cine MR images appeared as noise in the difference image. The true bladder V37Gy values were 15.98 and 16.27 cm^3^, whereas the predicted values were 11.01 and 11.95 cm^3^, showing notable discrepancies in these patients.

These results suggest that the larger differences between the PV‐MRI and true bladder V37Gy value may be associated with greater prediction errors. In Figure S4, a moderate positive correlation, with a Spearman's correlation coefficient of *r* = 0.66, between PV‐MRI and true bladder V37Gy, and the prediction errors were presented. Table 3 presents the results of the subgroup analysis that was conducted to identify the factors exhibiting larger prediction errors. A trend toward greater prediction errors was observed in patients with larger post‐MRI bladder volumes and greater volumes of bladder filling from PV‐ to post‐MRI; however, these differences were not significant (*p* = 0.099 and 0.076). In the subgroup of patients with posterior prostate movement, those with larger posterior movements exhibited greater prediction errors than those with smaller movements (*p* = 0.049). Additionally, patients with rectal gas had significantly greater prediction errors than those without (*p* = 0.020).

**TABLE 3:** Comparison of the prediction error (MAE) of Model 1 for predicting bladder V37Gy in each subgroup of the test dataset.

	Subgroup	No. of fractions	MAE (95% CI)	*p*‐value
PV‐MRI bladder volume (cm^3^)	< 140.29	56	1.19 (0.90–1.47)	0.149
	≥ 140.29	57	1.49 (1.17–1.80)	
Post‐MRI bladder volume (cm^3^)	< 201.4	56	1.2 (0.89–1.51)	0.099
	≥ 201.4	57	1.47 (1.18–1.77)	
Bladder filling (PV to post‐MRI) (cm^3^)	< 57.31	56	1.19 (0.89–1.49)	0.076
	≥ 57.31	57	1.48 (1.18–1.78)	
Risk group	Low	24	1.35 (0.90–1.80)	0.863
	Intermediate or High	89	1.33 (1.09–1.58)	
Anterior prostate movement (mm)	< 0.77	17	1.69 (1.02–2.36)	0.541
	≥ 0.77	18	1.85 (1.33–2.38)	
Posterior prostate movement (mm)	< 0.9	39	1.21 (0.94–1.49)	0.049
	≥ 0.9	39	1.85 (1.41–2.28)	
Superior prostate movement (mm)	< 0.55	17	1.8 (1.20–2.41)	0.804
	≥ 0.55	18	1.74 (1.14–2.34)	
Inferior prostate movement (mm)	< 0.63	39	1.41 (1.03–1.78)	0.242
	≥ 0.63	39	1.65 (1.28–2.03)	
Rectal gas	No	102	1.26 (1.04–1.48)	0.020
	Yes	11	2.07 (1.34–2.80	

Abbreviations: MAE: mean absolute error, V37Gy: volume of the bladder [in cm^3^] receiving a dose of ≥37 Gy, 95% CI: 95% confidence interval, PV: position verification, MRI: magnetic resonance imaging.

**FIGURE 7:**
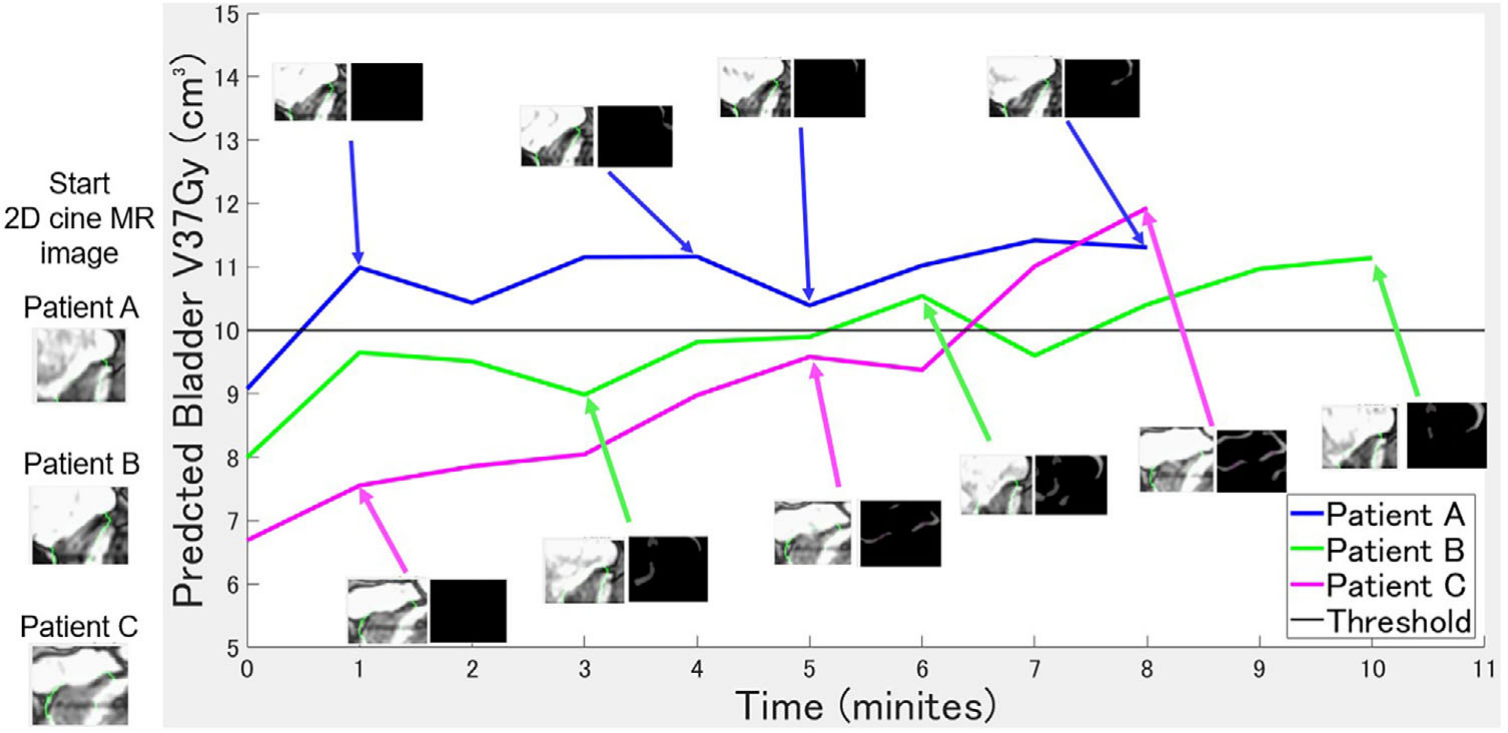
The line graph illustrating the trend in the predicted bladder V37Gy (volume of the bladder receiving ≥ 37 Gy) values derived from the mid‐irradiation images of three patients processed through Model 1 (main deep learning model). The *x*‐axis represents the elapsed irradiation time, and the *y*‐axis represents the bladder V37Gy values predicted by Model 1. The mid‐irradiation two‐dimensional (2D) cine magnetic resonance (MR) image and the corresponding difference images relative to the start 2D cine MR images are also shown. The black horizontal line indicates the threshold for the allowable bladder V37Gy dose constraint.

The results of predictions based on the 2D cine MRI data obtained during the mid‐irradiation period are shown in Figure 7. Although some variability was observed in the predictions, the predicted bladder V37Gy value exhibited an increasing trend in all three patients. This suggests that anatomical changes in the bladder are unlikely to be temporary and are more likely to occur progressively over time. Patients A, B, and C exceeded the bladder V37Gy threshold of > 10 cm^3^ in the early, middle, and late phases of irradiation, respectively.

Table 4 presents the predictive performance of the NasNetLarge and ResNet101 models for bladder V37Gy. In the training dataset, both models achieved significantly superior Spearman's correlation coefficients, MAE, and RMSE compared with Model 1. Contrarily, in the test dataset, both NasNetLarge and ResNet101 demonstrated a lower performance across all metrics, including Spearman's correlation coefficient, MAE, RMSE, AUC, and sensitivity, showing no improvement over Model 1.

**TABLE 4:** Spearman's correlation coefficient, MAE, RMSE, AUC, sensitivity, and specificity between the predicted and true bladder V37Gy in the training and test datasets for NasNetLarge model, ResNet101 model, and Model 1.

		NasNetLarge	ResNet101	Model 1	*p*‐value (NasNetLarge vs. Model 1)	*p*‐value (ResNet101 vs. Model 1)
Training	Spearman's correlation coefficient	1.00 ± 0.00	1.00 ± 0.00	0.99 ± 0.00	0.005	0.032
	MAE (cm^3^)	0.18 ± 0.02	0.19 ± 0.03	0.32 ± 0.03	0.002	0.008
	RMSE (cm^3^)	0.26 ± 0.04	0.27 ± 0.04	0.42 ± 0.03	0.003	0.007
	AUC	1.00 ± 0.00	1.00 ± 0.00	1.00 ± 0.00	0.006	0.229
	Sensitivity	0.96 ± 0.02	0.96 ± 0.02	0.95 ± 0.03	0.475	0.454
	Specificity	1.00 ± 0.00	1.00 ± 0.00	0.99 ± 0.00	0.008	0.330
Test	Spearman's correlation coefficient	0.83 ± 0.04	0.87 ± 0.02	0.89 ± 0.01	0.040	0.083
	MAE (cm^3^)	1.69 ± 0.15	1.53 ± 0.14	1.40 ± 0.05	0.031	0.138
	RMSE (cm^3^)	2.22 ± 0.21	2.03 ± 0.19	1.82 ± 0.06	0.014	0.044
	AUC	0.93 ± 0.04	0.95 ± 0.02	0.98 ± 0.00	0.045	0.012
	Sensitivity	0.60 ± 0.17	0.61 ± 0.20	0.91 ± 0.09	0.023	0.040
	Specificity	0.95 ± 0.01	0.95 ± 0.02	0.95 ± 0.01	0.670	0.871

Abbreviations: MAE: mean absolute error, RMSE: root mean squared error, AUC: area under the curve, V37Gy: volume of the bladder [in cm^3^] receiving a dose of ≥37 Gy.

The present study primarily focused on the prediction and analysis of bladder V37Gy; however, the prediction results for other OAR dose constraints are presented in Table 5. For reference, we also included the results obtained when using the PV‐MRI values alone for prediction. In the test dataset, the deep learning model demonstrated superior predictive performance compared with the predictions based on PV‐MRI for rectum V29Gy (*p* ≤ 0.001), rectum V18.1 Gy (*p* < 0.001), and bladder V18.1 Gy (*p* < 0.001), as evidenced by higher Spearman's correlation coefficients and lower MAE and RMSE values. However, the degree of improvement was limited. Specifically, for rectum V29Gy, the MAE values were 0.69 and 0.89 cm^3^ for the deep learning model and PV‐MRI‐based predictions, respectively. For rectum V18.1 Gy, the MAE values were 1.32 and 1.97 cm^3^, respectively, whereas, for bladder V18.1 Gy, the MAE values were 4.43 and 7.17 cm^3^, respectively.

**TABLE 5:** Spearman's correlation coefficient, MAE, RMSE between the predicted and true rectum V29Gy, V18.1 Gy, and bladder V18.1 Gy in the training and test datasets for Model 1 and prediction using PV‐MRI value.

			Model 1	Prediction using PV‐MRI value	*p‐*value
Rectum V29Gy	Training	Spearman's correlation coefficient	0.97 ± 0.00	0.62 ± 0.04	< 0.001
		MAE (cm^3^)	0.19 ± 0.03	1.04 ± 0.04	< 0.001
		RMSE (cm^3^)	0.26 ± 0.04	1.51 ± 0.07	< 0.001
	Test	Spearman's correlation coefficient	0.86 ± 0.01	0.79 ± 0.00	< 0.001
		MAE (cm^3^)	0.69 ± 0.04	0.89 ± 0.00	< 0.001
		RMSE (cm^3^)	1.67 ± 0.05	1.90 ± 0.00	0.001
Rectum V18.1 Gy	Training	Spearman's correlation coefficient	0.99 ± 0.00	0.77 ± 0.03	< 0.001
		MAE (cm^3^)	0.35 ± 0.06	2.23 ± 0.09	< 0.001
		RMSE (cm^3^)	0.46 ± 0.07	3.01 ± 0.09	< 0.001
	Test	Spearman's correlation coefficient	0.95 ± 0.00	0.89 ± 0.00	< 0.001
		MAE (cm^3^)	1.32 ± 0.04	1.97 ± 0.00	< 0.001
		RMSE (cm^3^)	2.19 ± 0.07	2.85 ± 0.00	< 0.001
Bladder V18.1 Gy	Training	Spearman's correlation coefficient	1.00 ± 0.00	0.92 ± 0.01	< 0.001
		MAE (cm^3^)	0.60 ± 0.07	4.99 ± 0.19	< 0.001
		RMSE (cm^3^)	0.81 ± 0.09	6.6 ± 0.22	< 0.001
	Test	Spearman's correlation coefficient	0.94 ± 0.00	0.91 ± 0.00	< 0.001
		MAE (cm^3^)	4.43 ± 0.15	7.17 ± 0.00	< 0.001
		RMSE (cm^3^)	6.14 ± 0.18	9.00 ± 0.00	< 0.001

Abbreviations: VXXGy: volume of the bladder [in cm^3^] receiving a dose of ≥XX Gy, MAE: mean absolute error, RMSE: root mean squared error, PV: position verification.

## 4 DISCUSSION

We developed deep learning models to estimate the OAR dose constraints (bladder V37Gy) from the 2D cine MRI and evaluated the accuracy of these models.

In the test dataset, the deep learning model showed improved Spearman's correlation coefficient, MAE, and RMSE as compared to approach using only the PV‐MRI bladder V37Gy (Table 1). Owing to the anatomical changes during irradiation, some sessions showed large discrepancies between the PV‐MRI and true bladder V37Gy values, appearing as outliers (Figure 5). The deep learning model was able to correct these values for such patients, indicating that it trained the anatomical changes as image features and predicted bladder V37Gy from the 2D cine MR image. However, the degree of improvement was limited (Table 1). Although the deep learning model was capable of capturing the anatomical changes during irradiation, its predictive accuracy was not yet optimal. The patients with a poor prediction accuracy of the model tended to have a substantial filling bladder, where even small anatomical changes led to large fluctuations in the bladder V37Gy value (Figure 6b). Furthermore, the subgroup analysis revealed that patients with substantial posterior prostate movements exhibited larger prediction errors than those with smaller posterior movements (Table 3). Bladder shape and volume vary considerably among patients; therefore, predictions based on a single sagittal plane struggled to accurately estimate the increase in bladder V37Gy caused by such large displacements. To further improve the predictive accuracy, including additional axial and coronal images or 3D spatial information through multi‐slices was necessary. Additionally, patients with rectal gas exhibited large prediction errors (Table 3). Only a few instances of such cases were included in the training dataset, suggesting that the model struggled to predict such edge cases accurately. The present study remains at an exploratory, proof‐of‐concept stage. Future work may enhance the clinical applicability of the approach by improving the model architecture, patient‐specific model adaptation, image preprocessing methods, and 2D cine MRI sequences, as well as by incorporating spatial information to increase the model's predictive accuracy and stabilize the methodology. Although the present study predicted bladder V37Gy at the end of irradiation, our approach can also predict bladder V37Gy during irradiation by inputting the 2D cine MRI data acquired during irradiation. Qualitative human assessment may overlook dose‐constraint violations because of the gradually occurring anatomical changes. Future deep learning‐based monitoring systems with improved accuracy could help detect‐dose constraint violations that might otherwise be missed by human observation alone.

Among the deep learning models, Model 1 showed good prediction accuracy in the test dataset (Table 2). Although Models 1 and 2 differed in their use of the difference image, both models exhibited comparable predictive performance (Table 2). No significant differences in the Spearman's correlation, MAE, RMSE, AUC, sensitivity, and specificity were observed between the two models, suggesting that the difference image was not a critical factor. Contrarily, the difference between Models 1 and 3 was the inclusion of the start and end 2D cine MR images, and Model 1 showed significantly better accuracy than Model 3 (Table 2). This indicates that the start and end 2D cine MR images provided clearer information about the shape of the MM, bladder volume, and extent of bladder overlap with the MM, which may have been obscured in the difference image alone. Models 4 and 5, which either excluded the PV‐MRI bladder V37Gy or replaced it with the pre‐MRI bladder V37Gy, also showed significantly lower accuracy than Model 1 (Table 2). This highlights the importance of the PV‐MRI bladder V37Gy as an essential predictor. However, calculating the PV‐MRI bladder V37Gy in actual clinical practice requires some manual effort, as the bladder contour must be transformed from the pre‐MRI to the PV‐MRI, followed by manual adjustments and dose recalculation. In our evaluation of five patients, the process took an average of approximately 2 min per case. Therefore, our approach necessitates resources either just before or immediately after starting irradiation to perform this process. Although the PV‐MRI bladder V37Gy can be obtained initially and subsequent estimations can be generated instantly using a deep learning model, the need for this preliminary manual step may still limit the practical feasibility of its true online clinical implementation. Therefore, an alternative approach to immediately obtain PV‐MRI bladder V37Gy are anticipated in the future.

In the present study, we employed a simple model architecture; however, no improvement in accuracy was observed even when using more complex architectures, such as ResNet101 and NasNetLarge (Table 4). This result may be partly due to model overfitting arising from the limited dataset size (381 samples from 91 patients), along with the simplicity of the prediction task (bladder V37Gy) and the restriction of input to sagittal images, which reduced the need for complex feature extraction. However, in the future, when using larger datasets or multiple imaging planes, more complex models with a greater number of parameters may be required for accurately prediction. The appropriate use of long short‐term memory models or hybrid architectures capable of handling time‐series data could also improve accuracy.

A sensitivity of Model 1 was 0.91 (Table 2). Model 1 could identify almost patients with bladder V37Gy of > 10 cm^3^. However, lowering the threshold allows for a further increase in sensitivity. When the threshold was set to the predicted bladder V37Gy of > 8 cm^3^, the sensitivity for detecting patients with bladder V37Gy of > 10 cm^3^ improved to 1.0. These results suggest that taking action when the predicted bladder V37Gy exceeds 8 cm^3^ may help prevent almost all dose constraint violations. For Patient A (Figure 7), patients who exceeded the dose constraint mid‐irradiation maintained elevated predicted values throughout the remainder of the treatment. This indicates that the anatomical changes in the bladder are not transient but occur progressively and persist over time. We visually identified patients whose bladder V37Gy increased substantially from PV‐ to post‐MRI, which was mostly due to posterior or inferior displacement caused by muscle relaxation or compression of the prostate due to bladder filling, with these anatomical changes persisting overtime. These observations were generally consistent with the predictions of the deep learning model during irradiation (Figure 7). For patients exceeding the bladder dose constraint during irradiation, a treatment plan shift is recommended for those with posterior or inferior movement of the prostate, whereas plan optimization should be performed for patients in whom bladder filling has caused bladder enlargement. However, pausing irradiation to implement an overall shift or replanning may prolong the overall treatment duration. For Patient C (Figure 7), where the dose constraint exceeded near the end of irradiation, the benefit of such interventions is limited. Therefore, it is important to implement corrective measures for patients exceeding the threshold early in the irradiation, as in Patient A (Figure 7).

The Unity MR‐Linac is equipped with the comprehensive motion management (CMM) function.^28^ One of the key features of the CMM is the “baseline shift,” which allows for adjusting the planned multileaf collimator positions during irradiation to align with the displaced target.^29^ This process can be completed within approximately 1–2 min.^30^ Therefore, by combining this functionality with our deep learning approach, prompt correction of the irradiation field position for patients predicted to exceed the OAR dose constraints, enabling rapid and efficient adaptation, may become possible in the future.

The present study primarily focused on the prediction and analysis of bladder V37Gy; however, from a clinical perspective, spatial dose information derived from multiple dose‐volume histogram (DVH) endpoints may be more informative. Thus, our approach was also applied to the prediction of rectum V29Gy, rectum V18.1 Gy, and bladder V18.1 Gy. Compared with the predictions using solely the PV‐MRI data, the predictive accuracy improved (*p* ≤ 0.001); however, the extent of improvement was still limited (Table 5). The bladder appeared with high signal intensity on 2D cine MRI, whereas the rectum was often difficult to visualize, which likely made it challenging for the deep learning model to accurately capture its anatomical boundaries. Additionally, for bladder V37Gy estimation, the MM region displayed on 2D cine MRI served as an approximate indicator of the area receiving the V37Gy dose. Contrarily, bladder V18.1 Gy represents a medium‐dose region, and, because our approach did not include information on the dose distribution as an input, our model may have had difficulty recognizing the spatial information of the medium‐dose area. In the present study, the deep learning model independently trained and predicted these DVH metrics. However, previous studies have demonstrated that simultaneous training on multiple tasks allows models to learn shared representations across related secondary tasks, resulting in a higher predictive accuracy compared to single‐task learning.^31^ Moreover, simultaneous training from multiple DVH endpoints may enable the model to better capture spatial dose distribution information. Therefore, in future work, simultaneous prediction of multiple DVH endpoints using this approach may further improve the predictive accuracy, not only for medium‐dose regions but also for high‐dose regions.

The present study has several limitations. First, only the PTV‐centered axial and sagittal planes were available for all prostate cancer patients. Since the bladder was not visible in the axial planes, only the sagittal planes could be used to estimate the bladder dose. Using only a single 2D plane cannot capture the full 3D anatomical information, likely contributing to the larger estimation errors in patients with large bladder volumes (Figure 6b). Consequently, improving accuracy will likely require incorporating multiple planes, including the coronal planes. Generally, 3D estimation approaches can utilize anatomical information from adjacent slices, leading to superior predictive accuracy in deep learning models compared to the 2D approaches.^32^ In the future, pseudo‐3D^33^ methods that enable estimation of 3D depth information of the bladder from 2D images or usage of multi‐planes may further improve the predictive accuracy. Second, the present study was limited to a single institution, single treatment site, and a single MR‐Linac system. To evaluate the generalizability of our study findings, larger multicenter datasets and external validation studies are required. Although our deep learning model predicted the dose constraints for the bladder and rectum, the high signal intensity of the bladder and SpaceOAR allowed our model to capture their changes. For other sites, such as the abdominal bowel, which lack high signal intensity on 2D cine MRI, predicting dose constraints may be more challenging. Furthermore, other MR‐guided radiotherapy systems, such as the ViewRay MRIdian (Oakwood Village, OH), with a lower magnetic field strength of 0.35 T and a larger voxel size of 3.50 mm × 3.50 mm^34^ might achieve lower predictive accuracy than the MR‐Linac system. Third, there was a time gap of a few minutes between the PV‐MRI acquisition and the start of irradiation and between the end of irradiation and post‐MRI acquisition. In the present study, we assumed that the 2D cine MRI at the start of irradiation corresponds to the PV‐MRI and that the 2D cine MRI at the end of irradiation corresponds to the post‐MRI. However, if anatomical changes have occurred during these time gaps, they may have negatively affected the model's training and predictive performance. Fourth, our approach could not produce a 3D dose distribution, which precluded deformation and accumulation of the dose distribution for accurate calculation of the dose delivered during irradiation. Obtaining an accurate dose calculation during irradiation would require reconstructing of the 3D MR images from the 2D cine MR images and incorporating log file‐based dose reconstruction methods.

## 5 CONCLUSION

We developed a deep learning model to estimate the OAR dose constraints from the 2D cine MRI acquired during irradiation and evaluated its performance. The predicted and true bladder V37Gy values in the test dataset showed a strong correlation (*r* = 0.89), and the sensitivity for detecting the dose constraint violations was 0.91. Nevertheless, the improvement of the predictions based solely on the PV‐MRI data was limited. Deep learning‐based estimation of OAR dose constraints during irradiation holds potential to assist clinical decision‐making for treatment interruption, plan adaptation, or positional adjustments. However, it is important to recognize the limitations inherent in these results.

## AUTHOR CONTRIBUTIONS

Shohei Tanakamainly drafted the manuscript. Noriyuki Kadoya and Keiichi Jingu reviewed the manuscript. Shohei Tanaka, Wingyi Lee, and Hisamichi Takagi performed the analysis. Yoshiyuki Katsuta, Kazuhiro Arai, Yushan Xiao, Taichi Hoshino provided guidance regarding planning and deep learning. Noriyoshi Takahashi provided clinical knowledge. All authors read and approved the final manuscript.

## CONFLICT OF INTEREST STATEMENT

Dr. Keiichi Jingu has rewarded a research grant from Elekta.

## ETHIC STATEMENT

The study protocol, data management procedures, and scientific rationale were approved by the institutional ethics committee of our hospital. Given that the present study was retrospective in nature involving no sample collection or intervention on human subjects, the requirement for patients’ informed consent was waived.

## Supporting information



Supporting Information

## Data Availability

It is difficult to public our dataset due to our hospital's policy.
