# Supplementary material for: Development and evaluation of deep learning models for estimating the organ at‐risk dose constraint from two‐dimensional cine magnetic resonance imaging scans during irradiation
Source: J Appl Clin Med Phys. 2025 Nov 27;26(12):e70403. doi: 10.1002/acm2.70403 (PMC12660054; doi:10.1002/acm2.70403)
Supplement: Supplementary file 1 — Supporting Information [file ACM2-26-e70403-s001.docx]

**Supplementary information**


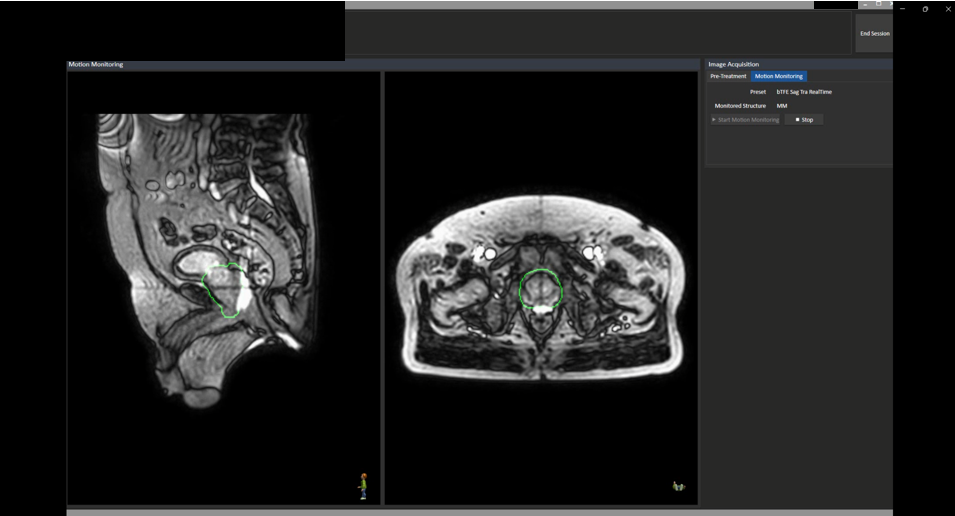


**Supplementary Figure S1.** Example of real-time two-dimensional (2D) cine magnetic resonance imaging (MRI) acquired during irradiation. The 2D cine MRI displayed on the screen was recorded, and a captured image from that recording is shown. The green line represents the motion monitoring (MM) area, which was defined to match the planning target volume in this study. Sagittal and axial images were obtained at the center of the MM area.


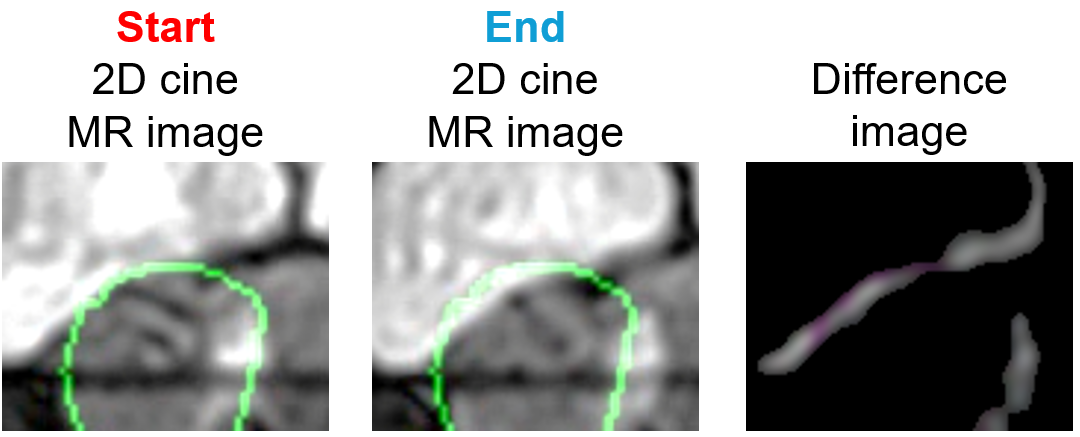


**Supplementary Figure S2**. Examples of two-dimensional (2D) cine magnetic resonance (MR) images used as inputs to the deep learning model. Sagittal images of a patient with prostate cancer are shown. From left to right: 2D cine MR image at the start of irradiation, 2D cine MR image at the end of irradiation, and the difference image between the start and end 2D cine MR images. The green line indicates the motion monitoring region, which corresponds to the planning target volume.

**
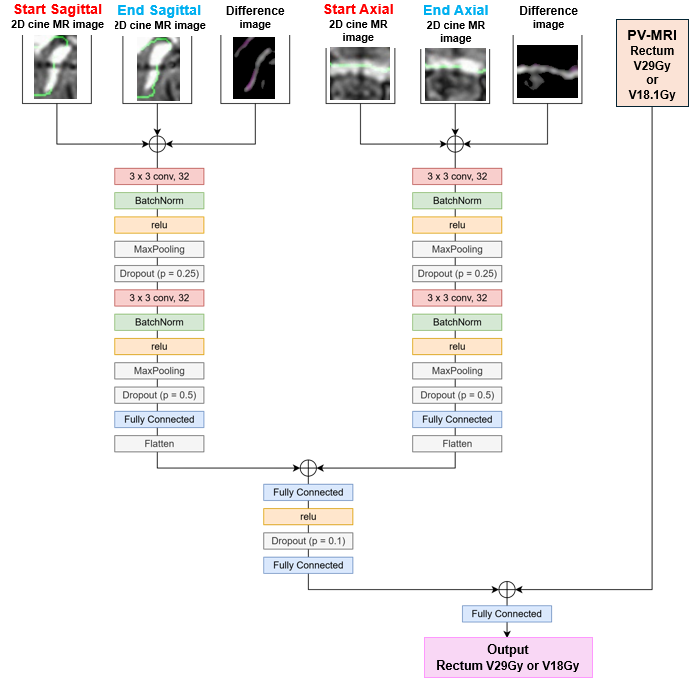
**

**Supplementary Figure S3.** Architecture of the deep learning model of predicting the volume of the rectum [cm^3^] receiving ≥29Gy (rectum V29Gy) or V18.1Gy from two-dimensional (2D) cine magnetic resonance imaging (MRI). The model takes 2D cine MR images and rectum V29Gy or V18.1Gy at the beginning of irradiation as inputs and outputs of the predicted bladder V29Gy or V18.1Gy at the end of irradiation.

**
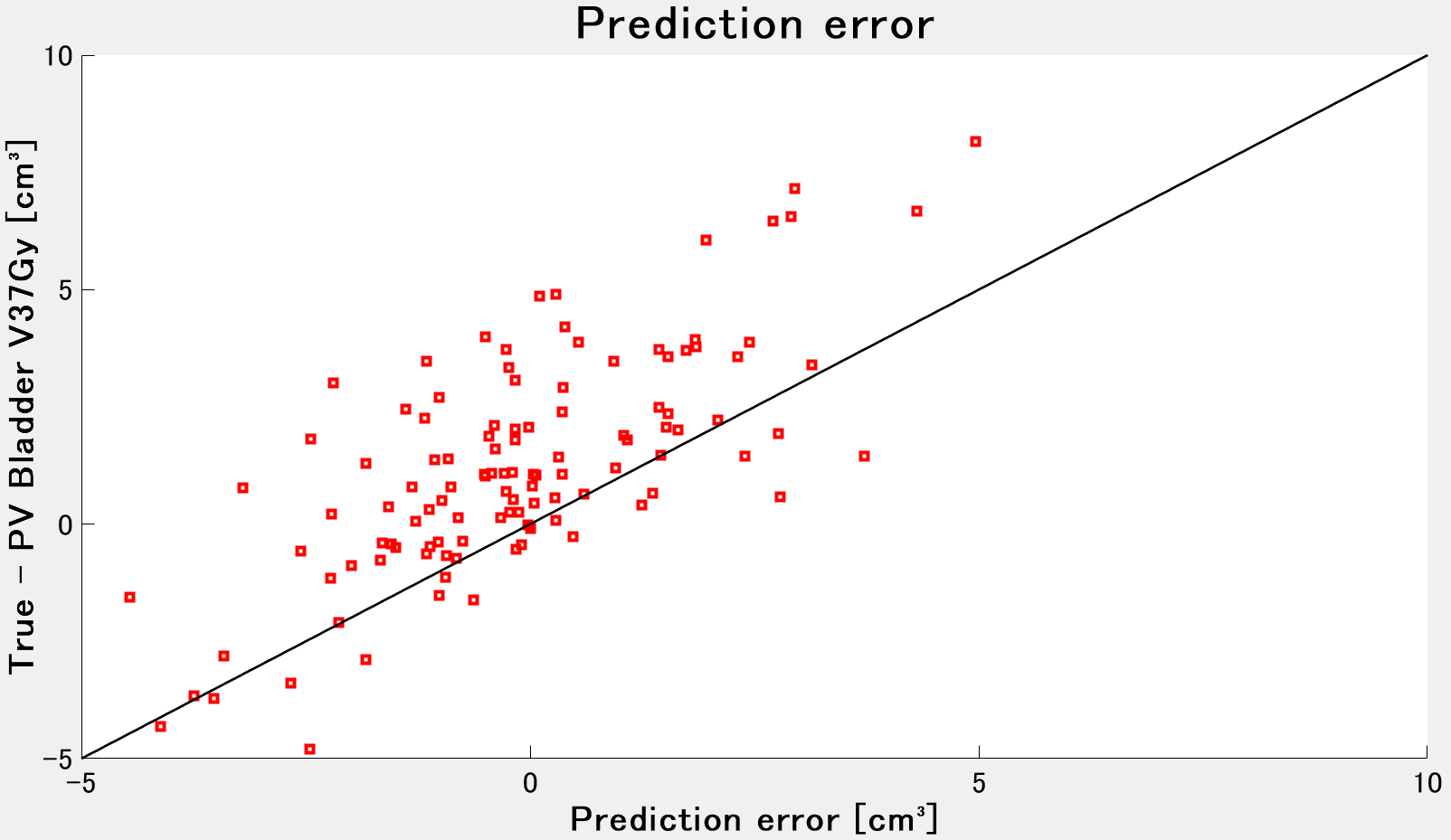
**

**Supplementary Figure S4.** Correlation between the prediction error (true bladder V37Gy−predicted V37Gy) of Model 1 in predicting the volume of the bladder [cm^3^] receiving ≥37Gy (bladder V37Gy) and the difference in bladder V37Gy between the post-magnetic resonance imaging (MRI) and position verification (PV)-MRI in the test dataset. In the present study, post-MRI bladder V37Gy is defined as true bladder V37Gy. The x-axis represents the prediction error, and the y-axis represents the difference in bladder V37Gy between the true and PV-MRI. A moderate correlation was observed (r = 0.66).

**Supplementary Table S1.** Dose constraints for low- and moderate-risk groups with prostate cancer

| Structure name |  | Optimal | Tolerable |
| --- | --- | --- | --- |
| PTV–rectum | D98% | >34.448 Gy | >32.625 Gy |
|  | D95% | 36.25 Gy |  |
|  | Dmax | <39.15 Gy |  |
| PTV and rectum overlap | D98% | >34 Gy | >32.625 Gy |
| Rectum | V36.0 Gy | <1 cc |  |
|  | V32.6 Gy | <15% |  |
|  | V29 Gy | <20% |  |
|  | V25.3 Gy | <30% |  |
|  | V21.7 Gy | <40% |  |
|  | V18.1 Gy | <50% |  |
| Bladder | V18.1 Gy | <40% |  |
|  | V37.0 Gy | <5 cc | <10 cc |
| Urethra PRV | V38.0 Gy | <0.1 cc |  |
| Femur head | V14.5 Gy | <5% |  |
|  | Dmax | <25.375 Gy |  |

DXX%: dose administered to XX% of volume, Dmax: maximum dose of the volume, VXXGy: cm^3^ or percentage of volume receiving a dose≥ XXGy, PTV: planning target volume, PRV: planning organ at risk volume

**Supplementary Table S2.** Dose constraints for the high-risk group with prostate cancer

| Structure name |  | Optimal | Tolerable |
| --- | --- | --- | --- |
| PTV–rectum–urethraPRV | D98% | >34.448 Gy | >32.625 Gy |
|  | D95% | 36.25 Gy |  |
|  | Dmax | <43.2 Gy |  |
| PTV and rectum overlap | D98% | >34 Gy | >32.625 Gy |
| Rectum | V36.0 Gy | <1 cc |  |
|  | V32.6 Gy | <15% |  |
|  | V29 Gy | <20% |  |
|  | V25.3 Gy | <30% |  |
|  | V21.7 Gy | <40% |  |
|  | V18.1 Gy | <50% |  |
| Bladder | V18.1 Gy | <40% |  |
|  | V37.0 Gy | <5 cc | <10 cc |
| Urethra PRV | Dmax | <40 Gy |  |
| Femur head | V14.5 Gy | <5% |  |
|  | Dmax | <25.375 Gy |  |
| CTV–urethraPRV | D95% | >100% | >98% |

DXX%: dose administered to XX% of volume, Dmax: maximum dose of the volume, VXXGy: cm^3^ or percentage of volume receiving a dose≥ XXGy, PTV: planning target volume, PRV: planning organ at risk volume, CTV: clinical target volume

**Supplementary Table S3.** Image cropping methods applied to 2D cine MR image as inputs for the deep learning models predicting dose constraints of the rectum and bladder.

|  | Preprocessing methods applied to 2D cine MR image as inputs for the deep learning model |
| --- | --- |
| Bladder | The 2D cine MR images were cropped from 30 pixels above to 60 pixels below the top of the motion monitoring (MM) in the cranio-caudal direction. The images were cropped from 55 pixels anterior to 55 pixels posterior to the center of the MM in the anterior–posterior direction. These regions were sufficient to cover the overlapping area of the bladder and MM. |
| Rectum | Sagittal 2D cine MR images were cropped from 30 pixels anterior to 30 pixels posterior the posterior to the most posterior motion monitoring (MM) boundary at the cranio-caudal central slice of the MM. The images were also cropped from 45 pixels above to 45 pixels below to the center of the MM in the cranial–caudal direction.  Axial 2D cine MR images were cropped from 30 pixels anterior to 30 pixels posterior the posterior of the motion monitoring (MM) in the anterior-posterior direction. The images were cropped from 35 pixels left to 35 pixels right to the center of the MM in the left–right direction.  These regions were sufficient to cover the overlapping area of the rectum and MM. |

2D: two-dimensional, MR: magnetic resonance

**Supplementary Table S4.** Architecture of the main deep learning model (Model1) for predicting the bladder V37 Gy from 2D cine MR image.

| Layer name | Output size | Kernel size | Padding size | Stride size | Dropout probability |
| --- | --- | --- | --- | --- | --- |
| Input (start 2D cine MR image) | 91 × 111 × 3 |  |  |  |  |
| Input (end 2D cine MR image) | 91 × 111 × 3 |  |  |  |  |
| Input (difference image) | 91 × 111 × 3 |  |  |  |  |
| Concatination_1 | 91 × 111 × 9 |  |  |  |  |
| Convolution_1 | 91 × 111 × 32 | 3 × 3 | (1,1) | (1,1) |  |
| Batch Normalization_1 | 91 × 111 × 32 |  |  |  |  |
| Relu_1 | 91 × 111 × 32 |  |  |  |  |
| Max Pooling_1 | 45 × 55 × 32 | 2 × 2 | (0,0) | (2,2) |  |
| Dropout_1 | 45 × 55 × 32 |  |  |  | 0.25 |
| Convolution_2 | 45 × 55 × 32 | 3 × 3 | (1,1) | (1,1) |  |
| Batch Normalization_2 | 45 × 55 × 32 |  |  |  |  |
| Relu_2 | 45 × 55 × 32 |  |  |  |  |
| Max Pooling_2 | 22 × 27 × 32 | 2 × 2 | (0,0) | (2,2) |  |
| Dropout_2 | 22 × 27 × 32 |  |  |  | 0.5 |
| Affine_1 | 1 × 1 × 20 |  |  |  |  |
| Flat_1 | 20 |  |  |  |  |
| Affine_2 | 20 |  |  |  |  |
| Relu_3 | 20 |  |  |  |  |
| Dropout_3 | 20 |  |  |  | 0.1 |
| Affine_3 | 2 |  |  |  |  |
| Input (PV-MRI bladder V37Gy) | 1 |  |  |  |  |
| Concatination_2 | 3 |  |  |  |  |
| Affine_4 | 1 |  |  |  |  |
| Regression Layer | 1 |  |  |  |  |

start 2D cine MR image: 2-dimensional cine magnetic resonance image at the start of irradiation, end 2D cine MR image: 2-dimensional cine magnetic resonance image at the end of irradiation, PV: position verification, V37Gy: volume of the bladder [in cm^3^] receiving a dose of ≥37Gy

**Supplementary Table S5.** Data augmentation methods and descriptions for the input data of the deep learning model.

| Input data | Augmentation methods and description |
| --- | --- |
| Start 2D cine MR image End 2D cine MR image | Random translation (±5 pixels in the X and Y directions), rotation (±5 degrees), brightness adjustment, and Gaussian noise were applied. |
| Difference image | Brightness adjustment and Gaussian noise were not applied because doing so would cause the overall pixel values of the difference images to become nearly zero. Instead, only random translation (±5 pixels in the X and Y directions) and rotation (±5 degrees) were applied. For the difference images corresponding to the start and end 2D cine MR image when brightness adjustment and Gaussian noise were applied, were copied from the original difference images. |
| PV-MRI bladder V37Gy | As the above image augmentation do not involve any changes to the original bladder V37Gy values, PV-MRI bladder V37Gy values corresponding to the augmented images were copied from the original values for data augmentation. |

2D: two-dimensional, MR: magnetic resonance, PV: position verification, V37Gy: volume of the bladder [in cm^3^] receiving a dose of ≥37Gy
